# Supplementary material for: Design of a Multi-Epitope Vaccine against Tuberculosis from Mycobacterium tuberculosis PE_PGRS49 and PE_PGRS56 Proteins by Reverse Vaccinology
Source: Microorganisms. 2023 Jun 24;11(7):1647. doi: 10.3390/microorganisms11071647 (PMC10385543; doi:10.3390/microorganisms11071647)
Supplement: Supplementary file 1 [file microorganisms-11-01647-s001.zip › microorganisms-2413547-supplementary.pdf]

Supplementary Material

Table S1. B cell epitopes identified with the IMED server of PE_PGRS49 and PE_PGRS56 proteins

Protein	Ubication	Sequence
PE_PGRS49	14-20	HGGPATN
	58-73	GSFGATSGPASIGVTG
	109-120	GSIGANSGIVGG
	132-138	GNGSLSS
	154-162	VGGNSSVTQ
	175-182	GGSGFFGG
	197-206	GGGTVGTVAG
	209-215	GNGGVGG
	217-225	GGDGVFAGA
	227-233	GQGGLGG
	276-284	GGGTQSATG
	301-307	SGAKAGG
PE_PGRS56	12-18	GDGGVGG
	26-34	NTTTAAAGT
	37-43	GAGGAGG
	81-91	GDGALAGSSGG
	101-113	DAGKAGTGSAPGT
	126-144	GGIGAAGTTGPVGTGASGG
	161-169	ANGGTAGAG
	181-190	GGAGVTSSTA
	204-219	GDAGAGGAGATPGANG
	228-242	GDGAAGAVGISGATG
	262-273	GAGGSGIDGVGG
	284-294	NGAIGGAGGDA
	314-320	GAGGAAG
	322-328	NGGTVGA
	339-346	GAAGAATA
	376-382	GIGGVGG
	389-401	ADGEVGGAGGAGG
	419-432	GSGGAGGAAGAGGA
	452-461	GGAGAASSAT
	488-495	GTGGAAGD
	500-516	GQGGAGGGAGGQGGAGG
	531-537	TAGTAGA
	576-589	GDRTVGGGTVPAGS
	600-609	GAGGQGGADG
	751-757	GDGGLTG
	783-789	NMTAQAG
	800-815	FGGGAGAGGGGLTAGA
	829-841	GNGAIGGHGPLTD
	859-868	GGAGIGSLGG
	885-891	EGGEVGG
	925-936	GTGGLGDPRVGG
	984-998	DAEPGVPPGAGGAGG
	1052-1060	DGGKAPAGG

Table S2. T cell epitopes identified with the Rankpep and Propred I servers of PE_PGRS49 and PE_PGRS56 proteins

Alleles	Epitopes sequence	
	PE_PGRS49	PE_PGRS56
HLA-A0101	(18-26) ATNPGSGSR<br>(320-328) GTEPGFGGD<br>(63-71) TSGPASIGV	
HLA_A0201	(110-118) SIGANSGIV	(832-839) AIGGHGPL.
HLA_A0301	(135-143) SLSSGEGGK	
HLA_A2402	(178-186) GFFGGKGGF<br>(128-136) GGAGGNGSL<br>(223-231) AGAGGQGGL<br>(240-248) GSTGGNGGL	
*HLA*B0702*		
HLA_B0801		
HLA_B1501		
*HLA*B3901*	(128-136) GGAGGNGSL<br>(223-231) AGAGGQGGL<br>(240-248) GSTGGNGGL<br>(277-285) GGTQSATGL	
HLA_B5801	(63-71) TSGPASIGV<br>(52-60) GGNGGDGSF<br>(109-117) GSIGANSGI<br>(171-179) AGGAGGSGF	
*DRB1*0101*		(781-789) TANMTAQAG<br>(1037-1045) WNGGKGDTG<br>(694-702) IAGMGGNGG<br>(811-819) LTAGANGTG<br>(527-535) ITGGTAGTA
*DRB1*0301*		
*DRB1*0401*	(222-230) FAGAGGQGG<br>(302-310) GAKAGGAGG<br>(274-282) GIGGGTQSA<br>(262-270) GFGGNGGKG<br>(278-286) GTQSATGLG<br>(225-233) AGGQGGLGG<br>(179-187) FFGGKGGFG	(29-37) TAAAGTTGG<br>(208-216) AGGAGATPG<br>(579-587) TVGGGTVPA<br>(159-167) GAANGGTAG<br>(339-347) GAAGAATAG<br>(780-788) NTANMTAGA<br>(433-441) GGGANGTAG<br>(996-1004) AGGAGTTGG<br>(102-110) AGKAGTGSA<br>(325-333) TVGANGTGG<br>(104-112) KAGTGSAPG<br>(22-30) NGADNTTTA<br>(128-136) IGAAGTTGP<br>(51-59) GGAAGTGTG<br>(537-545) AAGNGGAAG<br>(812-820) TAGANGTGG

		(333-GGAAGAATA
*DRB1*0701*		
*DRB1*0801*		
*DRB1*1101*	(263-271) FGGNGGKGG<br>(111-119) IGANSGIVG	(1036-1044) GWNGGKGDT<br>(645-653) GGANGGAGG<br>(585-593) VPAGSGGQG<br>(1034-1042) GTAWNGGKG
*DRB1*1301*		
*DRB1*1501*	(177-185) SGFFGGKGG	

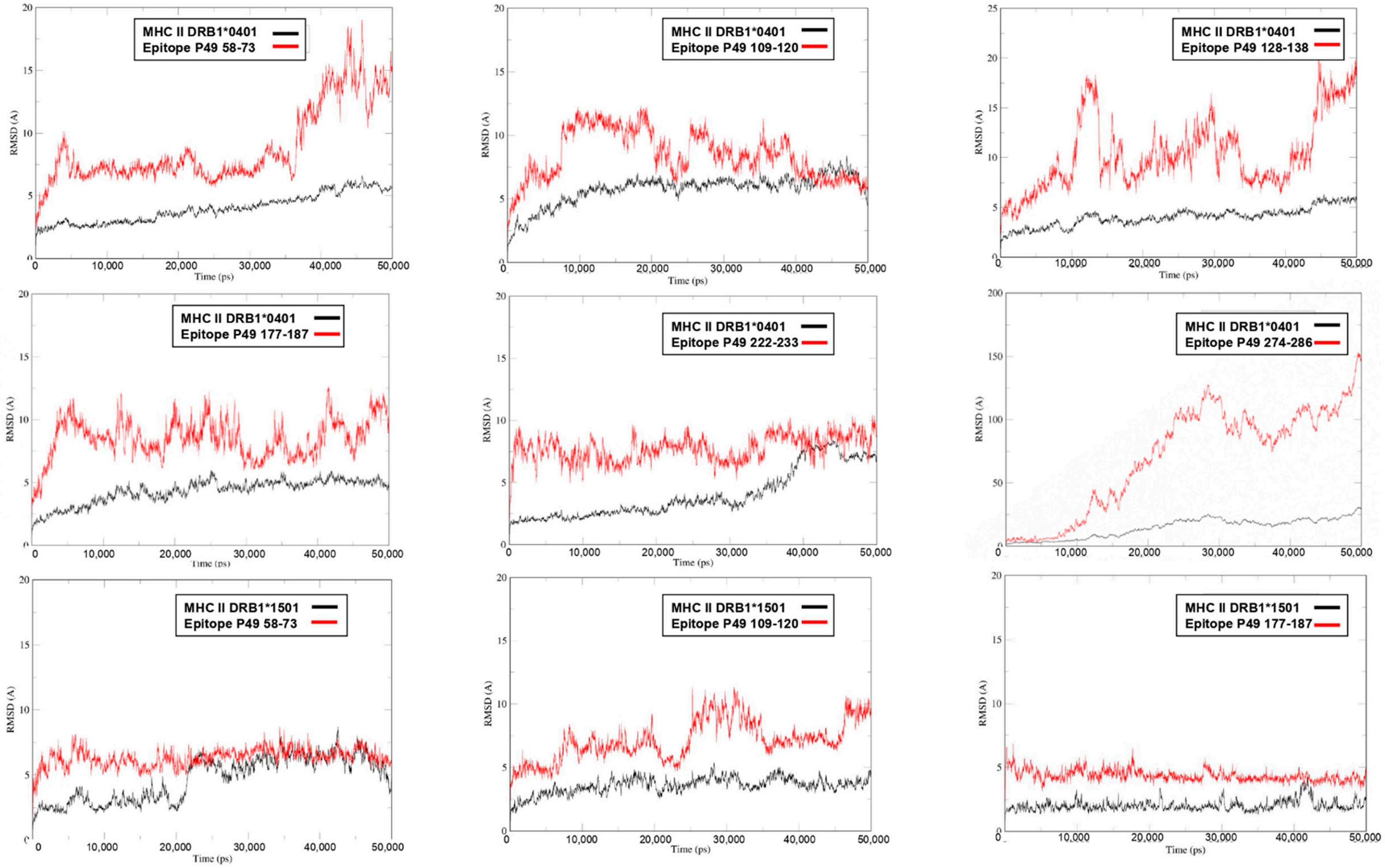

Figure S1. Molecular dynamics simulation of the epitope-receptor complex. The plots are showing the RMSD (X-axis = time in Frame and Y-axis = RMSD) of the epitopes PE_PGRS49 (red) and DRB1*0401 or DRB1*1501 (black). The most stable interaction is shown between the 222-233 and 177-187 epitope complex with DRB1*0401, The most stable interaction is shown between the 177-187 epitope complex with DRB1*1501.

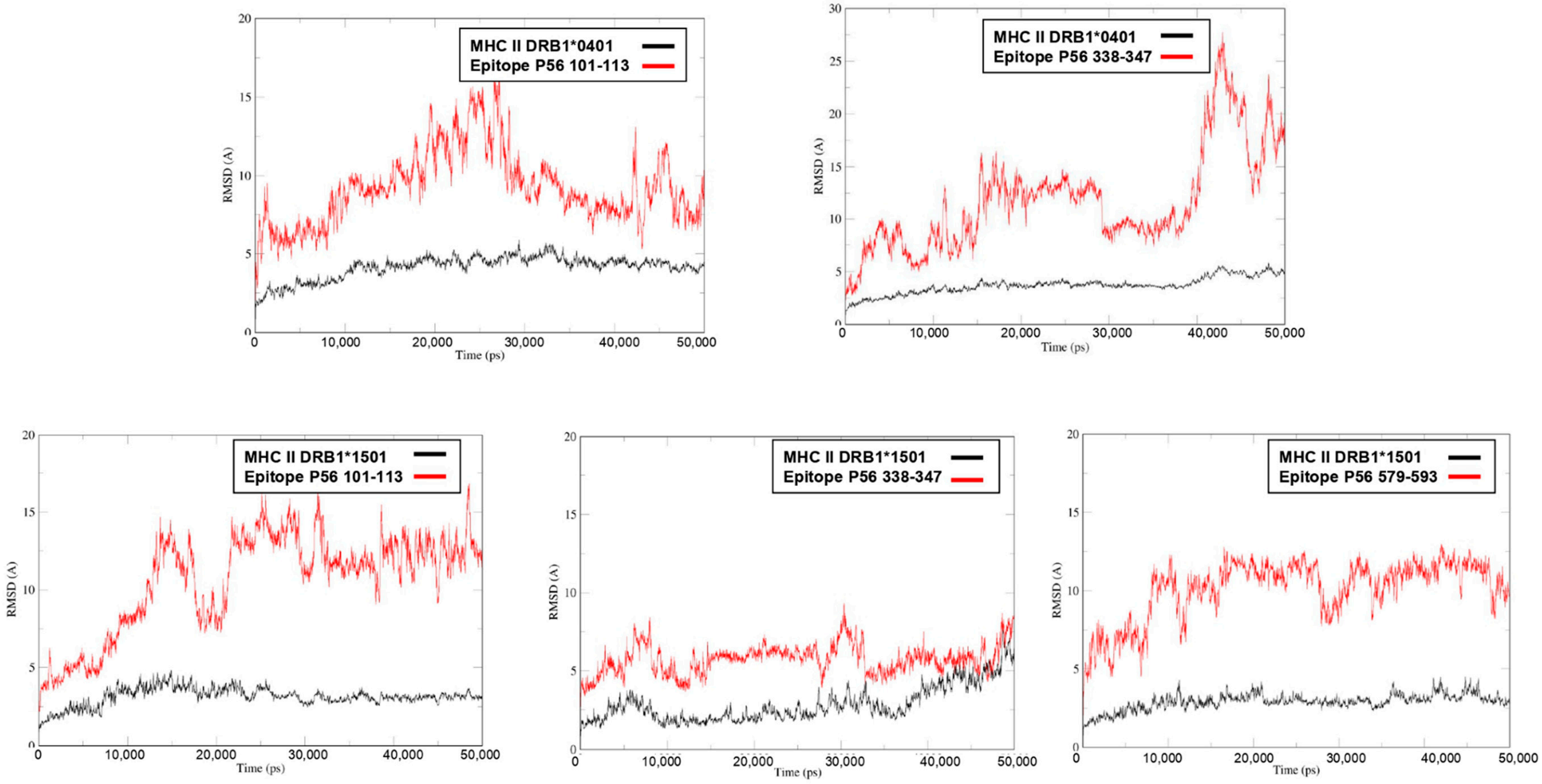

Figure S2. Molecular dynamics simulation of the epitope-receptor complex. The plots are showing the RMSD (X-axis = time in Frame and Y-axis = RMSD) of the epitopes (red) PE:PGRS56 and DRB1*1501 or DRB1*1501 (black). The most stable interaction is shown between the 338-347 epitope complex with DRB1*1501 and 579-593 epitope-DRB1*1501 showed an unstable interaction.

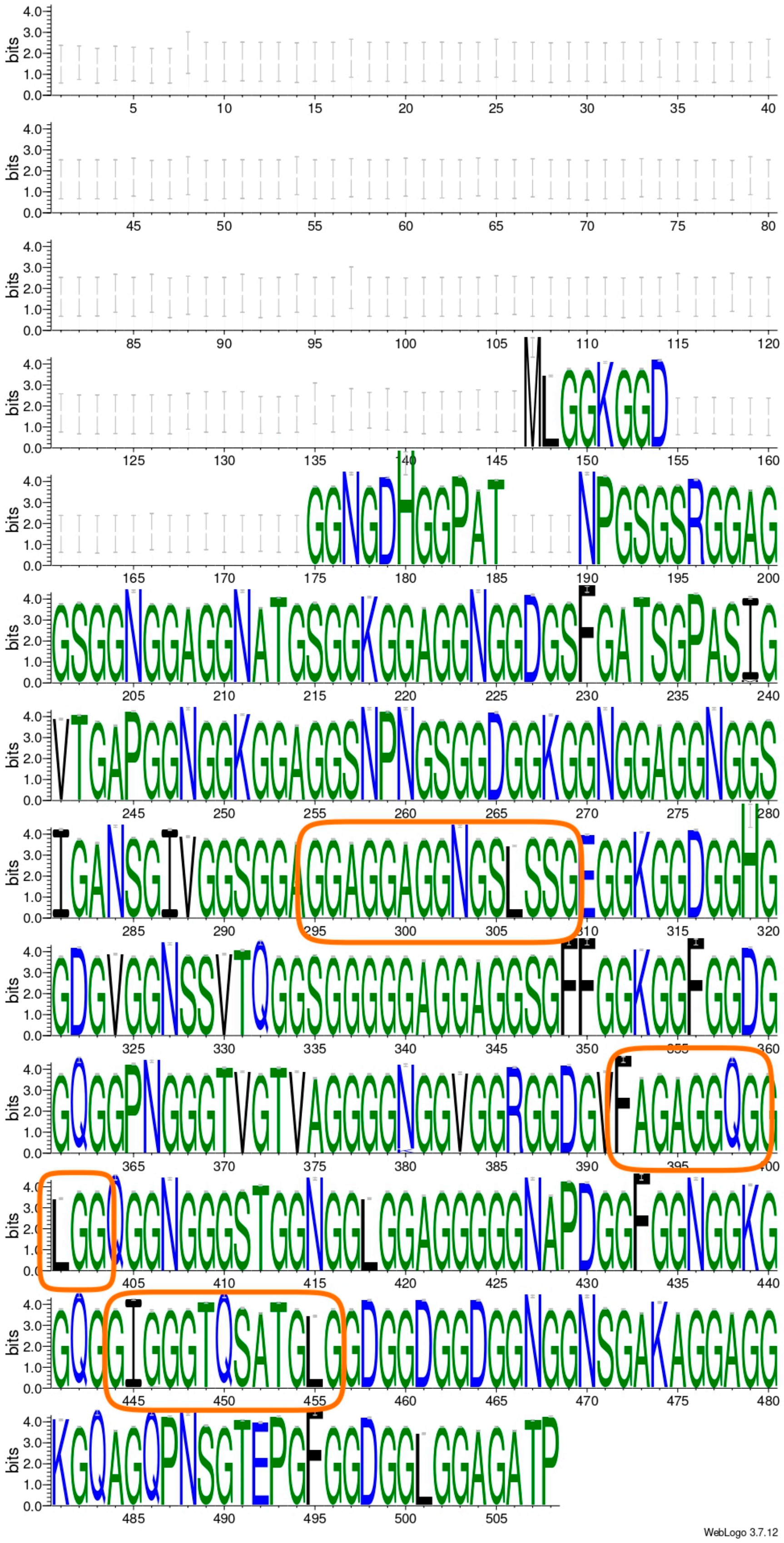

Figure S3. Graphic representation of the sequence alignment of the PE-PGRS49 protein. The red boxes indicate the location of the selected epitopes, all of which are in conserved regions.

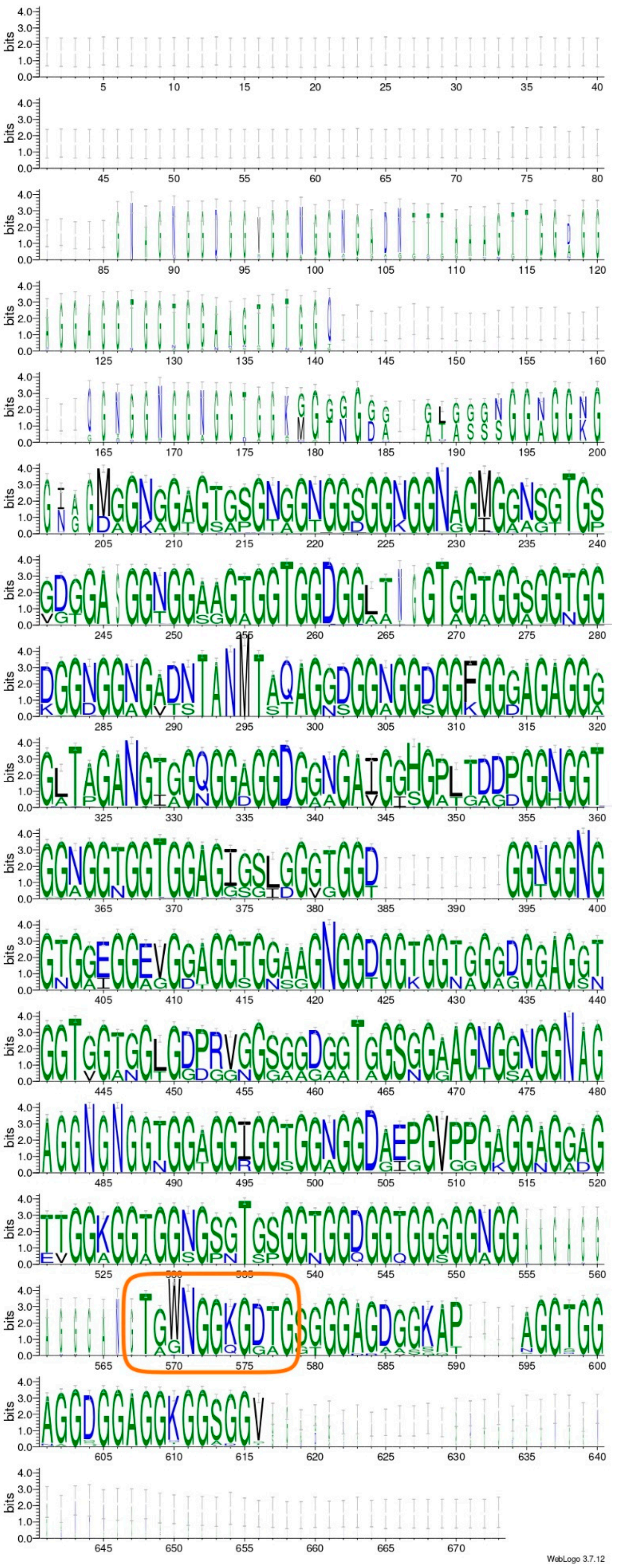

Figure S4. Graphic representation of the sequence alignment of the PE-PGRS56 protein. The red boxes indicate the location of the selected epitope, which is found in a conserved region.
